# National Retail Sales of Alcohol and Cannabis During the COVID-19 Pandemic in Canada

**DOI:** 10.1001/jamanetworkopen.2021.33076

**Published:** 2021-11-04

**Authors:** James MacKillop, Alysha Cooper, Jean Costello

**Affiliations:** 1 Peter Boris Centre for Addictions Research, St Joseph's Healthcare Hamilton/McMaster University, Hamilton, Ontario, Canada; 2 Michael G. DeGroote Centre for Medicinal Cannabis Research, McMaster University/St Joseph's Healthcare Hamilton, Hamilton, Ontario, Canada; 3 Homewood Research Institute, Guelph, Ontario

## Abstract

This economic evaluation examines national retail sales of alcohol and cannabis before and during the COVID-19 pandemic in Canada.

## Introduction

There is concern that the societal consequences of the COVID-19 pandemic will be associated with increased substance use.^1^ Data to date have primarily been self-reported changes, but objective sales data may inform this question. Here, we examined national retail sales of alcohol and cannabis prior to and during the pandemic in Canada.

## Methods

Where applicable, the report for this economic evaluation is consistent with the Consolidated Health Economic Evaluation Reporting Standards (CHEERS) reporting guideline. The Hamilton Integrated Research Ethics Board determined that ethical review board approval and informed consent were not needed because the data were publicly available sales metrics. The data were seasonally adjusted national monthly retail sales (ie, North American Industry Classification System codes 4453, for beer, wine, and liquor stores, and 453993, for cannabis stores) from November 2018 to June 2021 in Canadian dollars.^2^ The period was selected to provide a sizable prepandemic window and because of the timing of cannabis legalization (ie, mid-October 2018). Principal analyses were contrasts between intrapandemic sales and a counterfactual intrapandemic linear trend based on prepandemic sales. A subanalysis quantified stockpiling, operationalized as the proportionate change in March 2020, when states of emergency were declared, compared with the counterfactual estimate. Because the data were population level, null hypothesis significance testing was a secondary priority, but overall differences and intrapandemic trends in poststockpiling data were examined statistically using analysis of variance (ANOVA) and segmented regression, respectively. Significance tests used *P* < .05 and were 2-sided, and analyses were conducted from May to August 2021 using Excel version 2019 (Microsoft), SPSS statistical software version 26.0 (IBM), and R statistical software version 4.1.1 (R Project for Statistical Computing).

## Results

**Figure 1.**
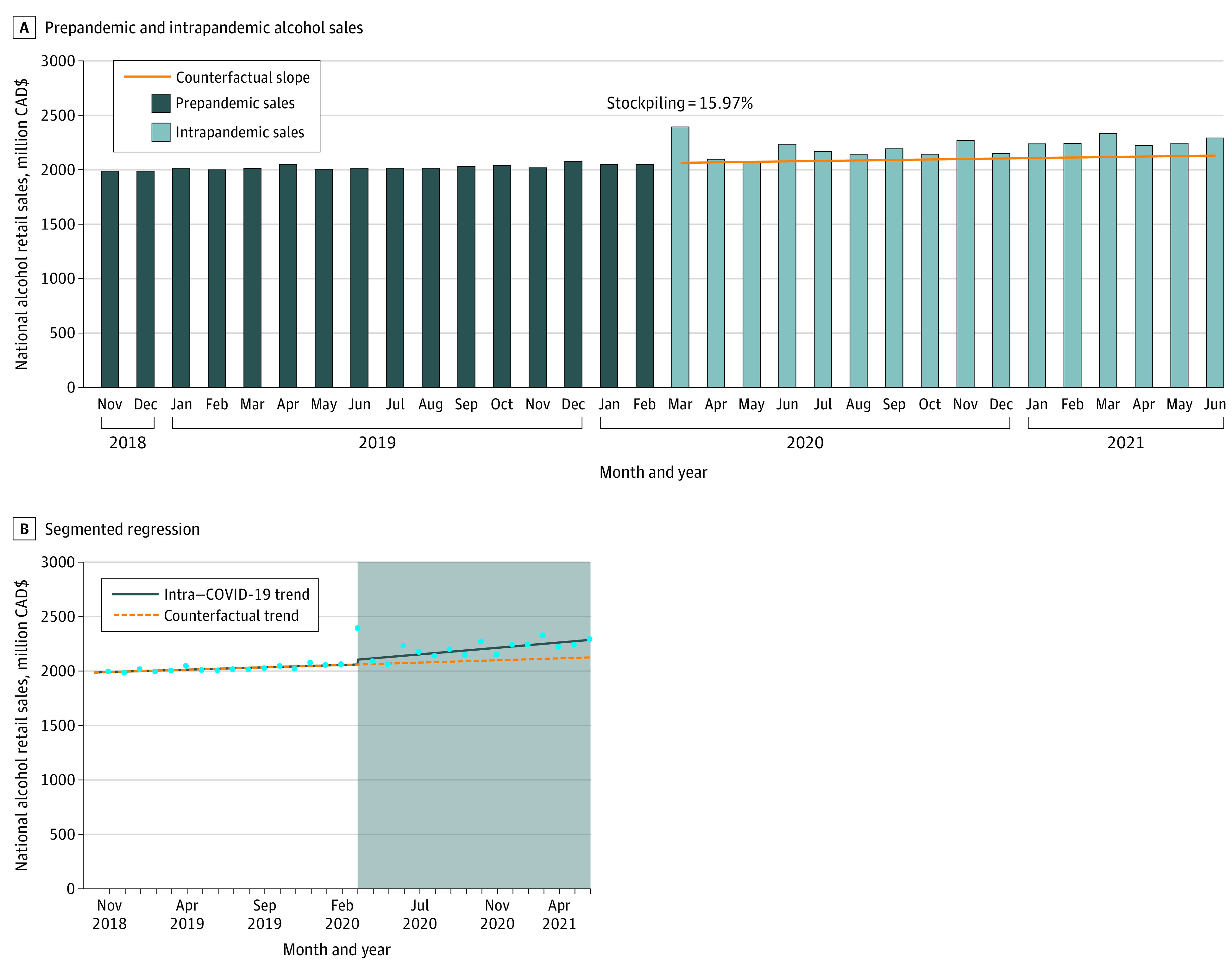
National Alcohol Retail Sales 16 Months Prior to and During the First 16 Months of the COVID-19 Pandemic in Canada A, Dark blue bars indicate prepandemic alcohol sales; light blue bars, intrapandemic alcohol sales; orange line, linear counterfactual trend. B, Circles indicate monthly sales; solid line, intrapandemic trend; dashed line, counterfactual trend; shaded area, COVID-19 period.

Mean (SD) monthly prepandemic alcohol sales were $2.02 billion ($26.24 million), exhibiting a positive slope of $4.45 million/month (Figure 1). Putative stockpiling in March 2020 was an increase of $330 million (15.97%) from the counterfactual estimate of $2.06 billion. Intrapandemic poststockpiling alcohol national monthly sales were $2.09 billion in April 2020 and $2.29 billion in June 2021. Mean (SD) monthly intrapandemic alcohol sales during the intra–COVID-19 period were $2.214 billion ($87 million), which was an increase of $116 million (5.54%) vs the mean (SD) counterfactual estimate of $2.098 billion ($21 million), reflecting an increase of $1.86 billion over the 16-month period. Based on ANOVA, mean monthly prepandemic sales were statistically significantly lower than intrapandemic sales ($2.02 billion; 95% CI, $1.99-$2.06 billion vs $2.21 billion; 95% CI, $2.18-$2.25 billion; *P* < .001). In segmented regression, there was an increasing trend in the time × COVID-19 interaction (*B* = 7.78; standard error [SE] = 3.07; *P* = .02) (Figure 1).

**Figure 2.**
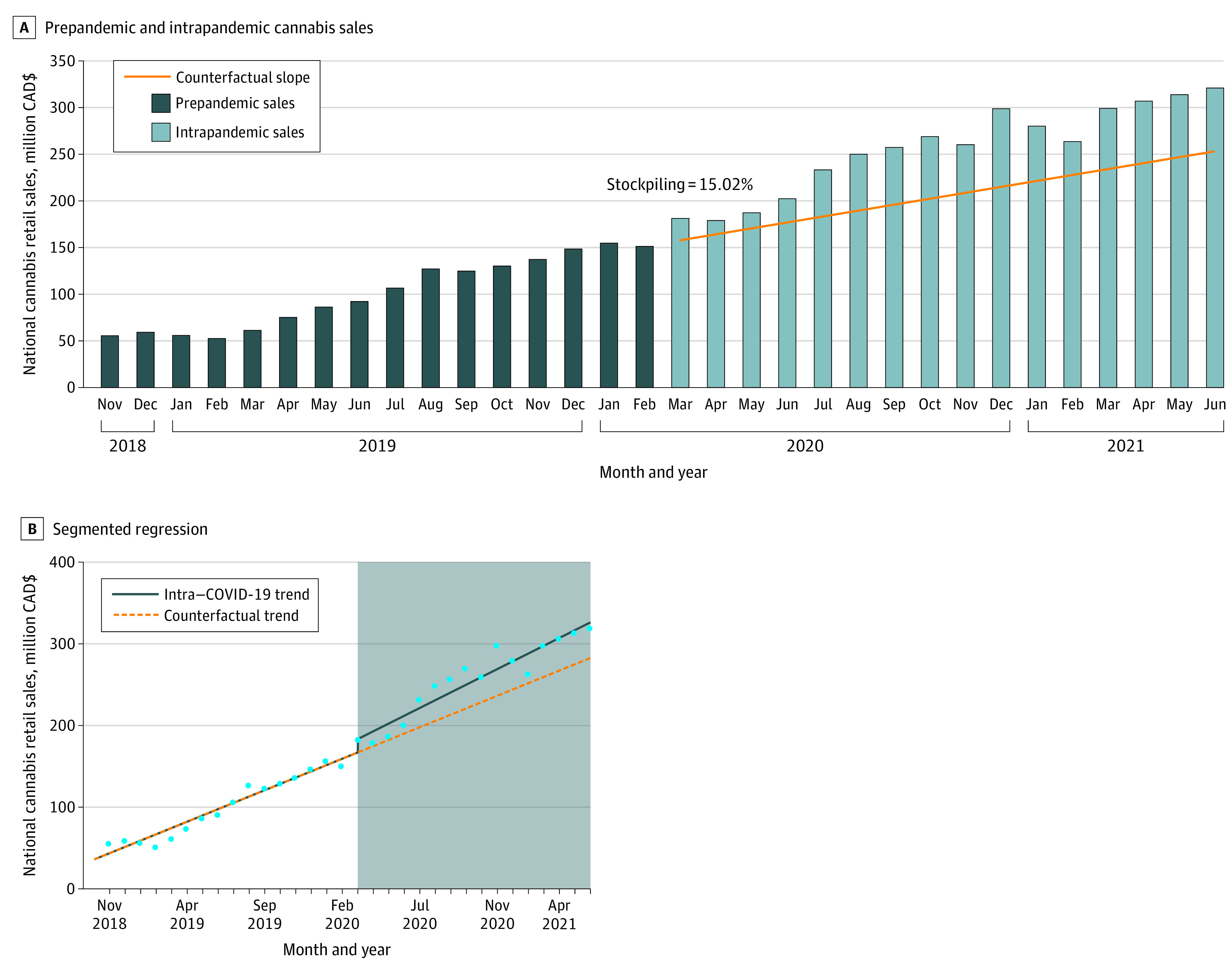
National Cannabis Retail Sales 16 Months Prior to and During the First 16 Months of the COVID-19 Pandemic in Canada A, Dark blue bars indicate prepandemic cannabis sales; light blue bars, intrapandemic cannabis sales; orange line, linear counterfactual trend. B, Circles indicate monthly sales; solid line, intrapandemic trend; dashed line, counterfactual trend; shaded area, COVID-19 period.

Monthly prepandemic cannabis sales exhibited an increase (Figure 2), from $55.40 million in November 2018 to $150.75 million in February 2020 (slope, $6.36 million/month), associated with the expanding legal marketplace. Stockpiling was estimated at an increase of $23.59 million (15.02%) greater than the counterfactual estimate of $157.10 million. Mean (SD) monthly intrapandemic cannabis sales were $255.51 million ($47.71 million), which was 24.78% greater than the mean (SD) counterfactual estimate of $204.78 million ($30.26 million) and reflected an increase of $811.74 million over the 16-month period. The difference in mean monthly prepandemic sales ($100.58 million [95% CI, $78.56-$122.61 million]) vs intrapandemic sales ($255.51 million [95% CI, $233.48-$277.54 million]) was significant (*P* < .001) but uninformative because of the steep postlegalization slope. In segmented regression, there was no statistically significant increasing trend (*B* = 1.82; SE = 1.43; *P* = .22) Figure 2).

## Discussion

In this economic evaluation, mean monthly national retail sales of alcohol, with notable stockpiling, exhibited a monthly increase of 5.5% vs the counterfactual estimate during the intrapandemic period. For cannabis, although stockpiling was similar, the general intrapandemic increase in mean monthly sales vs the counterfactual estimate was substantially higher, approaching 25%. Interestingly, these results converge with a national study of self-reported pandemic-associated changes that found a similar dissociation between alcohol and cannabis.^3^

The public health and clinical significance of these changes cannot be directly inferred. National retail sales cannot be directly converted into person-level expenditures, consumption behavior, or, more importantly, the extent to which individuals transition to higher-risk levels of use. Additionally, some subpopulations have reported decreases in alcohol involvement during the pandemic,^4^ and aggregate sales necessarily conflate increases, decreases, and stable patterns. A limitation of this study is that these macroeconomic indicators represent large proportions of the market but not its entirety (eg, illegal sales and alcohol sold through ferment-on-premises operations). This is particularly significant for cannabis, for which a large contraband market remains,^5^ and it is possible that the pandemic pushed consumers from illegal terrestrial purchasing to legal online purchasing.

These results nonetheless offer one of the first national perspectives on changes in alcohol and cannabis use during the COVID-19 pandemic. Whether similar patterns are present in other nations is an open question, but these findings suggest the value of sales data as a strategy to characterize the pandemic’s associations with substance use.
